# MicroRNA Regulation of Epigenetic Modifiers in Breast Cancer

**DOI:** 10.3390/cancers11070897

**Published:** 2019-06-27

**Authors:** Brock Humphries, Zhishan Wang, Chengfeng Yang

**Affiliations:** 1 Center for Molecular Imaging, Department of Radiology, University of Michigan, Ann Arbor, MI 48109; USA; 2 Department of Toxicology and Cancer Biology, College of Medicine, University of Kentucky, Lexington, KY 40536, USA; 3 Center for Research on Environment Disease, College of Medicine, University of Kentucky, Lexington, KY 40536; USA

**Keywords:** non-coding RNAs, microRNAs, epigenetics, breast cancer

## Abstract

Epigenetics refers to the heritable changes in gene expression without a change in the DNA sequence itself. Two of these major changes include aberrant DNA methylation as well as changes to histone modification patterns. Alterations to the epigenome can drive expression of oncogenes and suppression of tumor suppressors, resulting in tumorigenesis and cancer progression. In addition to modifications of the epigenome, microRNA (miRNA) dysregulation is also a hallmark for cancer initiation and metastasis. Advances in our understanding of cancer biology demonstrate that alterations in the epigenome are not only a major cause of miRNA dysregulation in cancer, but that miRNAs themselves also indirectly drive these DNA and histone modifications. More explicitly, recent work has shown that miRNAs can regulate chromatin structure and gene expression by directly targeting key enzymes involved in these processes. This review aims to summarize these research findings specifically in the context of breast cancer. This review also discusses miRNAs as epigenetic biomarkers and as therapeutics, and presents a comprehensive summary of currently validated epigenetic targets in breast cancer.

## 1. Non-coding RNAs (ncRNAs) and MicroRNAs (miRNAs)

The discovery that the majority of the human genome is not transcribed into protein [1], expanded our initial definition of a gene to include non-coding RNAs. Unlike the traditional definition of a gene, a DNA sequence that is transcribed into RNA then translated to produce a functional protein product, non-coding RNAs (ncRNAs) are functional RNA molecules that are not translated into protein. While the function of many of the ncRNAs in the genome is not yet determined, ncRNAs can generally be separated into two different groups: infrastructural and regulatory. Infrastructural ncRNAs are constitutively expressed and function within many cell homeostatic processes, such as splicing and translation, and include species such as transfer RNA (tRNA), ribosomal RNA (rRNA), small nuclear RNA (snRNA), and small nucleolar RNA (snoRNA). On the other hand, regulatory ncRNAs elicit effects on other RNA molecules, and can be further classified based upon their size into short (<200 nt), which includes microRNAs (miRNAs), small interfering RNAs (siRNAs), and Piwi-interacting RNAs (piRNAs), and long (lncRNA, >200 nt) ncRNAs.

**Figure 1:**
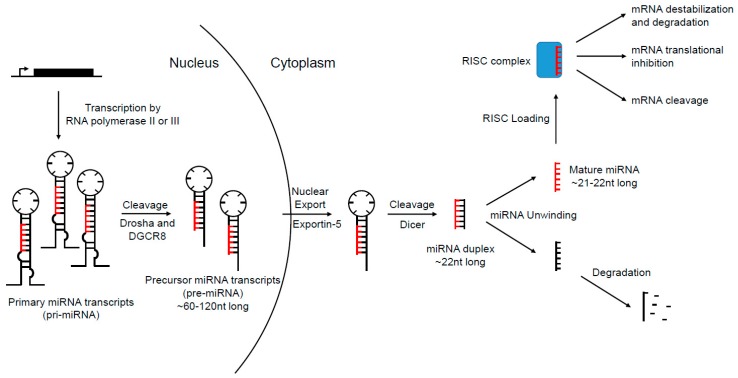
Canonical miRNA biogenesis pathway. miRNAs are transcribed in the nucleus via their own promoters or host gene promoters by RNA polymerase II or III, forming the primary miRNA transcripts which can range from hundreds to thousands of nucleotides long. Pri-miRNA transcripts are polyadenylated and capped, then subjected to a microprocessing cleavage event by an RNase III type enzyme, Drosha, and its binding partner DiGeorge syndrome critical region gene 8 (DGCR8) to form a 60–120 nucleotide long precursor miRNA transcript (pre-miRNA). After the cleavage event, pre-miRNAs are then exported out of the nucleus by exportin-5 to cytoplasm and again subjected to a microprocessing event by another RNase II enzyme, Dicer, to form a miRNA duplex. Unwinding of the miRNA duplex occurs and one strand is usually degraded, while the other is loaded into the RNA induced silencing complex (RISC). Once loaded, the RISC searches for targets of the miRNA in the genome. Once bound to a target mRNA, the RISC may induce negative expression of the mRNA by three ways: 1) mRNA destabilization and degradation, 2) mRNA translational inhibition, or 3) mRNA cleavage. The path at which the mRNA is regulated depends upon multiple factors of the mature miRNA.

MicroRNAs (miRNAs) are a large family of ~21–22 nucleotide RNAs that negatively regulate protein-coding gene expression post-transcriptionally in both metazoans and plants [2]. Canonically, miRNAs are transcribed in the nucleus by RNA polymerase II [3] or III [4] into primary miRNA (pri-miRNA), which range from hundreds to thousands of nucleotides long. Intronic miRNAs, located within a host gene, are transcribed using the same promoter as the primary transcript, while intergenic miRNAs rely on their own promoters [5,6,7]. After transcription by RNA polymerase, pri-miRNAs are then polyadenylated and capped. Pri-miRNA transcripts contain a stem-loop structure that contains the mature miRNA sequences, which are cleaved via an RNase III type enzyme, Drosha, and its binding partner DiGeorge syndrome critical region gene 8 (DGCR8) [8,9,10]. The resultant stem-loop intermediates are termed precursor miRNA (pre-miRNA). Each pre-miRNA is then exported to the cytosol via exportin-5 [11,12,13], and once in the cytosol is subjected to another microprocessing event carried out by the RNase III enzyme Dicer. Dicer cleaves the pre-miRNA into an RNA duplex (~22 nucleotides) containing the mature miRNA sequence [14,15,16,17,18]. In most cases, one strand of this duplex is preferentially degraded, while the other is selected to be loaded into the RNA induced silencing complex (RISC) [19,20]. Once loaded into the RISC, the mature miRNA provides the binding specificity for the complex, and the complex elicits the negative regulation of the target mRNA. A figure graphically describing the canonical miRNA biogenesis pathway is shown in Figure 1. It should also be noted that recent research has identified several miRNAs that are formed by bypassing key steps in the canonical miRNA biogenesis pathway [21,22]. However, these non-canonical miRNAs still function like the canonical miRNAs.

Although exceptions have been found, miRNAs typically elicit their inhibitory effects by base pairing with the 3′ untranslated regions (3′-UTR) of target mRNAs through their seed sequence. The seed sequence is the second to eighth nucleotide region at the 5′ end of the mature miRNA, and is primarily responsible for determining the specificity of the miRNA to its mRNA. Although the seed sequence is critical for miRNA-loaded RISC recognition of target mRNA, the overall miRNA:mRNA complementarity also impacts binding efficiency [2,23]. For example, a recent study demonstrated that the 3′ end of the miRNA itself is critical in determining target recognition in vivo [24]. In addition, many of the sites that match the miRNA seed sequence in the 3′UTR are preferentially conserved [25], suggesting conserved regulatory functions for miRNAs. Once bound, miRNA base pairing can drive mRNA destabilization and degradation, translational inhibition, or direct mRNA cleavage [26,27,28,29]. While mRNA destabilization and degradation as well as translation inhibition are both common, direct mRNA cleavage occurs in rare cases and requires more extensive base pairing [28,29].

Since miRNAs can target multiple genes and can share their seed sequence with other miRNAs, it is predicted that miRNAs can target over half of the human genome [5,25,30]. Therefore, miRNAs are implicated in almost all cellular functions. An increasing wealth of knowledge now exists linking miRNA dysfunction to cancer initiation and progression [31,32,33]. Since epigenetics has recently emerged as an important factor in driving cancer initiation and progression, microRNAs likely are involved in regulating the DNA and epigenetic machinery. Indeed, Dicer-specific ncRNA (miRNA and/or siRNA) is necessary to form heterochromatin [34] and accumulate heterochromatin-specific proteins [35], and a growing body of evidence demonstrates that miRNAs directly target and negatively regulate important epigenetic machinery in breast and other cancers. We understand that miRNA expression has been shown to be regulated by epigenetic mechanisms and this is reviewed elsewhere [36,37]. The findings from current studies strongly suggest that miRNAs only indirectly regulate epigenetics by regulating chromatin- and histone-modifying enzymes. This review will focus on the epigenetic machinery and the miRNAs that have been shown to target this machinery in breast cancer.

## 2. The Epigenome and Its Regulators

Epigenetics refers to the heritable changes in gene expression without a change in the DNA sequence itself [38]. Rather, they depend on the alteration of other properties such as DNA methylation, histone modification patterns and ncRNAs, which are inherited through cell divisions. Although most histone modifications have not yet been shown to be meiotically heritable, some studies suggest that in males, histone-bearing nucleosomes are retained throughout spermatogenesis and therefore some epigenetic modifications may be preserved across generations [39,40,41]. Epigenetic modifications can broadly be classified into the following groups, and a table of known epigenetic regulators can be found in Table 1.

### 2.1. DNA Methylation and Demethylation

In mammals, the primary epigenetic tag found on DNA is a stable but reversible, covalent attachment of a methyl group to the C-5 position of the cytosine ring, converting it to a 5-methylcytosine (5mC) [42]. The predominant target for DNA methylation are clusters of CpG nucleotides, which are frequently associated with gene promoters [43], and methylation of promoter CpGs is almost always associated with gene silencing. However, DNA methylation can also occur in the gene body leading to increased gene expression in dividing cells [44,45]. In normal cells, DNA methylation predominantly occurs in highly repetitive genomic regions [46] and a balance is important for normal development and functioning. However, dysregulation of methylation drives aberrant gene expression and can contribute to disease. Indeed, dysfunctional hypo- and hypermethylation of genes is a frequent and early event in breast cancer, and correlates with disease severity and outcome [47,48,49]. 

#### 2.1.1. DNA Methyltransferases

DNA methylation is regulated by a family of DNA methyltransferases (DNMTs), of which three catalytically active DNMTs have been identified in mammals: DNMT1, DNMT3a, and DNMT3b. DNMT3a and DNMT3b are both thought to be responsible for establishing the pattern of methylation de novo [50]. Due to their de novo activity, much of the research on DNMT3a/b and miRNAs focuses on regulation of DNMT3a/b downstream regulated targets. However, miRNA direct targeting of DNMT3a/b has revealed controversial results in cancer progression, with some showing oncogenic [51] and others a tumor suppressor function [52,53]. Additionally, it has been shown that miR-148 directly targets only one of the three splice variants of DNMT3b (Dnmt3b1) [54], suggesting that splice variant-specificity of miRNAs is a field of research that needs to be further explored. Although DNMT1 also has de novo activity [55], DNMT1 typically thought of as required for maintaining the pattern of DNA methylation during DNA replication [56,57]. Methyl-binding domain (MBD) proteins are the primary candidates for the readout of DNA methylation [58,59] because of their ability to bind to methylated CpG sites, and are thought to induce gene silencing by recruiting chromatin remodelers, histone deacetylases, and methylases to methylated DNA [60,61].

#### 2.1.2. DNA Demethylases

Despite its stability, DNA methylation can be reversed and this can occur as either an active or passive process [62]. Passive DNA demethylation can occur when there is a lack of functional DNA methylation machinery, which results in the failure to keep 5mC tags on the newly synthesized DNA strand and thus dilutes 5mC following replication. On the other hand, active DNA demethylation is mediated by the ten eleven translocation (TET) family of proteins: TET1, TET2, and TET3. TET proteins facilitate DNA demethylation by two different mechanisms: i) physical binding to DNA to prevent unwanted DNA methylation [63,64], and ii) enzymatic removal of the methyl group from 5mC. The mechanism for demethylation includes multiple oxidation steps which are all mediated by TET proteins: i) oxidation of 5mC to 5-hydroxymethylcytosine (5hmC), ii) oxidation of 5hmC to 5-formylcytosine (5fC), and iii) oxidation of 5fC to 5-carboxylcytosine (5caC) [65,66,67]. Although dilution of these demethylation intermediates can occur through replication, a demethylated cytosine typically occurs through thymine DNA glycosylase (TDG)-mediated base excision followed by base excision repair (BER) of 5caC and 5fC [68,69]. Generally, miRNAs that target the TET family of DNA demethylases act as oncogenic miRNAs (oncomiRs) [70,71,72], likely due to the reactivation of silenced oncogenes. Additionally, some evidence exists demonstrating that DNMT3a and DNMT3b also are important in DNA demethylation of promoters [73,74], suggesting an increased complexity, and a possibility of DNMT targeting miRNAs, in the mechanism of DNA demethylation.

### 2.2. Histone Modifications

**Figure 2:**
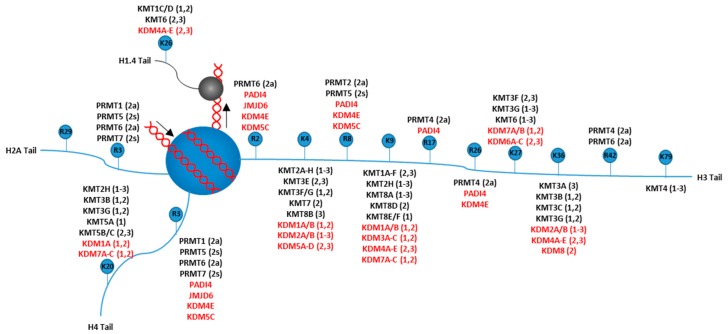
Writer and erasers for histone methylation. A schematic representation of a nucleosome and the known principal lysine (K) and arginine (R) residue methylation sites on the H1.4, H2A, H3, and H4 tails. The number associated with the lysine or arginine residue represents that residues location on the depicted tail (i.e., “K5” on histone H2A tail refers to the lysine residue at amino acid 5 on the H2A tail). The writers (lysine or arginine methyltransferases, black text) and the erasers (lysine and arginine demethylases, red text) for each methylation site are also shown with their known methylation state specificities: monomethylation: 1; dimethylation: 2; trimethylation: 3; asymmetrical dimethylation: 2a; symmetric dimethylation: 2s. Arrows near the DNA indicate direction that the DNA wraps around the histone.

**Figure 3:**
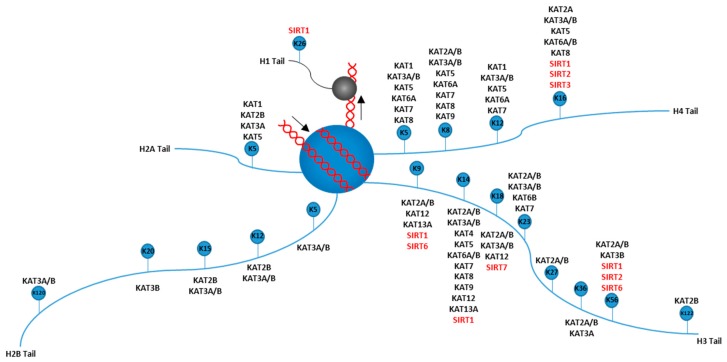
Writers and erasers for histone acetylation. A schematic representation of a nucleosome and the known principal lysine (K) acetylation residue sites on the H1, H2A, H2B, H3, and H4 tails. The number associated with the lysine residue represents that residues location on the depicted tail (i.e., “K5” on histone H2A tail refers to the lysine residue at amino acid 5 on the H2A tail). The writers (lysine acetyltransferases, black text) and the erasers (lysine deacetylases, red text) for each methylation site are also shown. Arrows near the DNA indicate direction that the DNA wraps around the histone.

Chromatin architecture may be transmissible to daughter cells [75], therefore histones and modifications to histones are likely candidates for carriers of epigenetic information. Histones are the protein that DNA is wrapped around within chromatin, which consists of an octomeric globular core (H2A, H2B, H3, and H4) and these cores are connected to each other by a short stretch of linker DNA, which gives the “beads on a string” structure. The linker histone H1 binds to the entry and exit sites of DNA from the histone core, which stabilizes higher-order chromatin structures [76]. Each monomer of the core, as well as the H1 linker histone, contains a tail which can be targeted for histone modifications. Histone modifications are covalent post-translational modifications to histone proteins which alters chromatin structure or recruits histone modifiers. Histone modifications that regulate epigenetics include methylation and acetylation, which are typically accompanied by the epigenetic regulators of the trithorax (TrxG) and polycomb (PcG) group of proteins [77,78]. These epigenetic modifications coordinate DNA accessibility, and thus regulate gene expression throughout development and in breast cancer. Therefore, it is thought that histone methyl- and acetyl-modifying enzymes represent promising therapeutic targets in breast cancer [79,80,81]. Figures showing which histone tail residues are acetylated and methylated, and which enzyme performs each modification is shown in Figure 2 and Figure 3.

#### 2.2.1. Histone Methyltransferases

In addition to DNA methylation, histones can also be methylated. Assessment of genome-wide histone methylation marks show that lysine and arginine residues are particularly enriched for methylation, and whereas DNA methylation is associated with silencing, histone methylation is correlated with both silencing and activation [82]. The two major families of histone methyltransferases (HMTs) are lysine methyl transferases (KMTs) and protein arginine methyltransferases (PRMTs) [83], which methylate their respective residues.

Histone lysine residues can either be mono-, di-, or trimethylated and are catalyzed by two main KMT families: SET domain containing methyltransferases and the DOT1 family [84]. One of the most well-known KMTs is KMT6A (also known as EZH2). EZH2 is highly abundant in proliferative cells, and establishes repressive marks on PcG target genes to exert its oncogenic potential [85]. Due to its well-known role in oncogenesis, many studies have shown that miRNAs can directly target and negatively regulate KMT6A expression to abrogate its effects on cancer progression [86,87,88,89]. 

In contrast to histone lysine residues, histone arginine residues can only be mono- or dimethylated [84]. These PRMTs can also be divided into type I and type II PRMTs, where type I PRMTs form mono- and asymmetric dimethylarginines and type II PRMTs form mono- and symmetric dimethylarginines [84]. Although both KMTs and PRMTs methylate histone tail residues, most research has focused on miRNA regulation of KMTs. Research to date generally supports the fact that miRNAs act as tumor suppressors by decreasing KMT expression.

#### 2.2.2. Histone Demethylases

Histone demethylation is carried out by two different classes of histone demethylases (HDMs): amine-oxidase type lysine-specific demethylases (LSDs) and the JumonjiC (JMJC) domain containing histone demethylases. Although both of these classes remove methyl groups from histones, they differ in their catalytic mechanism. LSD demethylases are flavin adenine dinucleotide (FAD)-dependent allowing these to remove methyl groups from mono- and di-, but not trimethylated lysines [84]. While JMJC domain containing demethylases are iron and 2-oxoglutarate (2-OG)-dependent enzymes, and can demethylate all three methyl lysine states as well as arginine residues [84,90]. The specificity of these HDMs for methyl groups is often determined by its incorporation within different complexes. Demethylation of targets by HDMs typically results in enhanced expression of genes that drive proliferation, migration, and invasion [91,92,93,94], which suggests that miRNA regulation of these proteins is important in inhibiting cancer cell survival and progression.

#### 2.2.3. Histone Acetyltransferase

Histone acetylation is central in regulation of DNA accessibility for transcription [95,96]. Acetylation of histones primarily occurs on the histone tails and controls gene expression through two mechanisms: i) direct effect on the stability of the histone:DNA complex and ii) creating docking sites for regulatory complexes [97,98]. Two groups of histone acetyltransferases (HATs) exist, type A and type B. Type A HATs acetylate histones and other chromatin-associated proteins in the nucleus, and type B HATs acetylate newly synthesized histones in the cytosol [99,100]. Although both types acetylate histones, type B HATs have no direct influence on transcription [100]. Type A HATs can be split into five families: GNAT, MYST, p300/CBP, nuclear receptor coactivators, and general transcription factors. Since most HATs exist within multiprotein complexes, it is the context in which the HATs exist that determines the acetylation specificity, and these complexes are determined upstream through changes within the cellular environment. Much of the work on miRNA regulation of HATs has focused on two specific HATs: the NCOA (KAT13) family, nuclear transcriptional coactivators, and p300 (KAT3B), a well-known tumor suppressor [101]. Therefore, miRNA regulation in HAT-driven processes has elucidated both oncogenic and tumor suppressor functions for miRNAs. 

#### 2.2.4. Histone Deacetylases

Histone deacetylases (HDACs) are devoted to the removal of acetyl groups from lysine residues. HDACs exist in multi-unit protein complexes and are typically thought to repress transcription. Based upon their structural homology to yeast HDACs, human HDACs can be divided into four classes: Class I Rpd3-like proteins, Class II Hda1-like proteins, Class III Sir2-like proteins, and a single Class IV protein [102]. Although divided into separate classes, class I, II and IV share a conserved zinc ion-mediated catalytic mechanism to hydrolyze the bond of the acetylated lysine. However, Class III HDACs use NAD^+^ as a reactant to deacetylate lysine residues. In addition to interactions with histones, HDACs can also deacetylate non-histone proteins in breast and other cancers [103,104,105]. As in HAT regulation, the role that miRNAs play in HDAC-mediated cellular functions yield both oncogenic and tumor suppressor miRNAs. Although a majority of the research has yielded important functions of miRNAs in HDAC-mediated drug resistance.

## 3. MicroRNAs Regulate the Machinery of the Breast Epigenome

In addition to being subjected to epigenetic regulation themselves through DNA and histone modifications of their corresponding genes [36,37], miRNAs also play a more decisive role in chromatin structure control and gene expression by directly targeting the post-transcriptional regulation of key chromatin- and DNA-modifying enzymes. This subclass of miRNAs are also known as epi-miRs [106], and can affect the epigenetic enzymes either directly or indirectly. This section will focus on miRNAs that have been identified to directly bind to the 3′UTR of epigenetic machinery in breast cancer. However, a full list of breast epi-miRs that have been shown to reduce epigenetic machinery mRNA and/or protein expression is shown in Table 2.

### 3.1. Targeting of DNA Methyltransferases

Since DNA methylation is the primary epigenetic tag in mammals, it is not surprising that miRNAs have been found to directly target the enzymes that catalyze these tags. For example, Ng et al. found that miR-143 directly targets DNMT3A in breast cancer [52]. Mechanistically, direct targeting of DNMT3A by miR-143 results in a decrease in promoter methylation-specific silencing of the tumor suppressors phosphatase and tensin homolog (PTEN) and tumor necrosis factor (TNF) receptor superfamily member 10c (TNFRSF10C). This results in decreased growth and anchorage-independent colony formation of breast cancer cells [52]. 

Although most well known for their role in regulating epithelial-mesenchymal transition (EMT) [33], the miR-200 family also can regulate the methylation machinery to regulate triple negative breast cancer (TNBC). Since the expression of the miR-200 family is suppressed in TNBC, Pang and colleagues aimed to understand the epigenetic mechanisms underlying miR-200b repression in TNBC [53]. They first found an inverse correlation between DNMT3A and miR-200b in TNBC breast tissues, which suggests epigenetic modification of miR-200b expression. Indeed, both treatment of TNBC cells with 5-aza-2′-deoxycytidine (DAC, a demethylating agent) and knockdown of DNMT3A expression increased miR-200b expression by alleviating promoter methylation. They also found that silencing of miR-200b is mediated by MYC binding to the promoter region of miR-200b and recruiting DNMT3A. Interestingly, miR-200b was also found to directly target DNMT3A [53], suggesting a complex feedback loop between DNMT3A and miR-200b. Overall, this feedback loop may be critical for understanding the regulation of EMT in TNBC, and suggests that epigenetic silencing of the miR-200 family is important in TNBC initiation and progression.

In addition to regulating growth and EMT through DMNT3A, epi-miRs can regulate stemness of breast cancer cells. Roscigno and colleagues found that the epi-miR, miR-221, regulates the stemness of breast cancer cells by directly targeting DNMT3B [51]. They first found that in breast cancer stem cells (BCSCs) isolated from patients, miR-221 was significantly upregulated compared to differentiated cells. Furthermore, enriching multiple breast cancer cell lines for stem cells in vitro not only resulted in an increase in stemness markers, but also resulted in increased miR-221 expression, suggesting that miR-221 is involved in regulating BCSCs. Indeed, overexpressing miR-221 increased, and inhibiting decreased, mammosphere formation, stemness marker expression, and enrichment of the CD24^−/low^/CD44^+^ stem cell population. Since stemness gene expression is primarily regulated by methylation, this group also looked at the role of miR-221 in methylation. Interestingly, overexpression of miR-221 reduces methylation of the CpG islands located within the promoters of stemness genes *Nanog* and *Oct3/4*. Mechanistically, Roscigno et al. determined that miR-221 directly targets DNMT3B, and expression levels of DNMT3B is inversely associated with miR-221 expression [51]. Together, this data demonstrated that miR-221 contributes directly to silencing of DNMT3B, leading to the aberrant expression of stemness genes.

### 3.2. Targeting of DNA Demethylases

MicroRNAs also target DNA demethylases, mediated by the ten eleven translocation (TET) family, which can lead to dysregulation of genes involved in critical cellular functions. Two members of the miR-29 family (miR-29a [71] and miR-29b [72]) have independently been shown to directly target TET1 in breast cancer. In addition to targeting TET1, both of these studies demonstrated that expression of the miR-29 family member promoted proliferation and EMT in vitro. However, in breast tissues miR-29a expression was found to be increased in ER(−) samples [71] and miR-29b decreased in all samples [72] compared to normal adjacent tissue. This discrepancy may be due to separation of samples by subtype in the previous study and no separation in the latter study. Additionally, it is not uncommon to have members of the same family differentially expressed in different cancer types and even within the same cancer [33,142], which adds complexity to our understanding of these disease states.

In addition to the miR-29 family, miR-22 has also been shown to directly target TET1 [70]. Stable expression of miR-22 in normal breast and breast cancer cells enhanced migration and induced EMT both in vitro and in vivo settings. As the miR-200 family is involved in regulating EMT, Song and colleagues next looked at whether miR-22 could affect the expression of the miR-200 family members. They found that miR-22 could repress miR-200a and miR-200c expression in both normal mammary epithelial cells and mouse mammary epithelium. This is accomplished by miR-22 stimulating promoter hypermethylation through targeting of TET family members. Although a luciferase reporter assay was only performed for TET2, miR-22 was also shown to regulate TET1 and TET3 mRNA and protein levels [70]. This demonstrates that miR-22 functions as an epigenetic modifier and promotes EMT, stemness, and metastasis in breast cancer. 

### 3.3. Targeting of Histone Methyltransferases

Enhancer of zeste homolog 2 (EZH2), is the catalytic component of the polycomb repressive complex 2 (PRC2) and is highly expressed in breast cancer. Due to its well-known role in many cell fate decisions [143], many microRNAs have been shown to directly target EZH2 to modulate its expression in breast cancer [86,87,89,121,126,133]. In addition to changes in cell proliferation, anchorage-independent growth, migration, invasion, autophagy and apoptosis, reduction of EZH2 expression concomitantly reduced global H3 methylation levels. Interestingly, stable expression of three of these miRNAs (miR-101, miR-138, and miR-340) not only reduced EZH2, but also affected the expression of epigenetically regulated miRNAs [121,126,133]. However, in all cases reduction of EZH2 by miRNAs inhibited tumor growth and metastasis in mouse models, suggesting that these miRNAs act as tumor and metastasis suppressors, and demonstrate that EZH2 is an important therapeutic target in breast cancer.

In breast cancer, both SETDB1 and SETD8 are directly targeted by miR-7 [107,108]. Expression of miR-7 was shown to suppress EMT in both more differentiated and breast cancer stem cells. Zhang et al. demonstrated that by targeting SETDB1, the expression of the SETDB1 downstream effector STAT3 is also reduced [107]. Since, STAT3 promotes the expression of TWIST and TWIST is physically associated with SETD8 [144], miR-7 seems to regulate multiple epigenetic regulators within the same pathway. Additionally, miR-7 promotes DNA damage and STAT3 activation is cytoprotective, which suggests that miR-7 also regulates apoptosis through binding to epigenetic regulators. Therefore, miR-7 is heavily involved in regulating multiple pathways involved in breast cancer progression.

### 3.4. Targeting of Histone Demethylases

MiRNAs also directly regulate the expression levels of histone demethylases (KDMs) in breast cancer. The main member of the amine-oxidase type lysine-specific demethylases, KDM1A (also known as LSD1), is a direct target of miR-708 in triple negative breast cancer [91]. Increased expression of miR-708 in MDA-MB-231 cells resulted in a reduction in cell growth and invasion, which was rescued by stable expression of KDM1A. Furthermore, Shao et al. also found that KDM7B (or PHF8) has oncogenic effects on breast cancer cells [115]. Expression of KDM7B promoted mammosphere formation and EMT by reducing the methylating marks near the transcription start site of *SNAI1*. However, stable expression of miR-22 was able to abrogate these phenotypes and regulate the expression of KDM7B. In addition to KDM1A and KDM7B, many members of the Jumonji (JMJC) domain containing family are also directly targeted by miRNAs through binding sites in their 3’UTR; miR-491-5p targets KDM4B (JMJD2B) [136], miR-137 targets KDM5B (JARID1B) [94], and miR-138 targets KDM5C (JARID1C) [94]. Overall, these studies demonstrate that each of the miRNAs elicit tumor suppressor effects through direct binding and inhibition of histone demethylase expression.

### 3.5. Targeting of Histone Acetyltransferases

Different studies have shown that the histone acetyltransferase KAT13B (NCOA3) is targeted by multiple miRNAs in breast cancer [110,111,112]. The study by Eedunuri and colleagues not only demonstrated that KAT13B protein expression is modulated by miR-137, but also that miR-137 reduced protein expression of the other family members KAT13A (NCOA1) and KAT13C (NCOA2) suggesting that miR-137 targets multiple members of the KAT13 family [112]. KAT13B can act as a coactivator for nuclear receptors (such as the estrogen receptor) which can modify the chromatin environment to facilitate gene expression. Consistent with this, inhibition of KAT13B by miR-17-5p was not only able to severely limit ER-dependent cell growth, but it also reduced IGF-1-mediated growth [111]. Additionally, increased expression of KAT13B can also modify chromatin to drive expression of chemoresistance to common therapeutics such as taxol [110]. These studies suggest that loss of miRNA regulation can promote epigenetic mechanisms that feed cancer cell progression.

In addition to targeting the coactivators of nuclear receptors to change chromatin structure and gene expression, miRNAs also modulate histone acetyltransferases to epigenetically regulate EMT downstream effectors. Recent works demonstrated that the miR-106b-25 cluster (miR-106b, miR-93, and miR-25) and miR-22 target KAT3B [118] and KAT5 [114], respectively, in breast cancer. However, in contrast to the above studies, by reducing levels of KAT3B and KAT5, these miRNAs promote the expression of EMT markers and drive cell migration, invasion, and stemness. This suggests that, at least for histone acetyltransferases, miRNA involvement in epigenetic mechanisms that control cancer progression may be context and target dependent.

### 3.6. Targeting of Histone Deacetylases

HDAC protein levels are also modulated by several miRNAs in breast cancer. Both miR-34a and miR-10b are involved in epigenetic regulation of treatment response of breast cancer by targeting HDACs [109,119]. Wu and colleagues demonstrated that miR-34a does this, at least in part, by inhibiting the deacetylation of lysine 246 on heat shock protein 70 (HSP70) [119]. Additionally, two separate studies found that miR-125a-5p directly targets HDAC4 [122] and HDAC5 [123] to inhibit multiple biological functions of breast cancer cells, such as apoptosis, growth, and motility. It was also concluded that miR-125a-5p may have an application as a prognostic biomarker as serum levels of miR-125a-5p correlated with tumor stage and lymph node status [122].

## 4. MicroRNA as Epigenetic Biomarkers and Therapeutic Targets

The epigenetic status of a tumor can strongly influence its behavior and aggressiveness [145,146,147,148], suggesting that epigenetic markers can relate to clinical prognosis. Since the symptoms of a tumor often do not present themselves until the primary tumor has progressed to invade the surrounding tissue and because epigenetic changes occur very early in the transition to disease states, the use of epigenetic biomarkers may provide an avenue for early detection and cancer prevention. Current efforts to utilize epigenetic markers as a biomarker focus on histone and promoter methylation as a source for prognostic information and predictive power [149]. Although miRNAs have been demonstrated as highly tissue-specific biomarkers [150,151,152], the use of miRNAs as epigenetic biomarkers has been less explored. Still, some studies have identified miRNA signatures containing some known epi-miRs that correlate with patient outcome in breast cancer as well as overall risk, receptor expression, tumor stage, and proliferation index [153,154,155,156]. Furthermore, single nucleotide polymorphisms (SNPs) in the epi-miR binding site of the target mRNA contributing to early breast cancer development [157] demonstrates that miRNAs show promise as epigenetic biomarkers.

In the clinic there are two strategies for epigenetic therapy: i) small molecules that inhibit the epigenetic machinery (DNA methyltransferase inhibitors (DNMTi) and histone deacetylase inhibitors (HDACi). The treatment with DNMTi and/or HDACi has proven to be very beneficial for hematological malignancies by turning on tumor suppressors that were silenced, but has controversial results for solid tumors [158,159]. And ii) the manipulation of miRNA expression. Several direct and indirect strategies are used to silence oncogenic miRNA and to express tumor suppressor miRNAs [160], and the main advantages of using miRNAs as epigenetic therapeutics include the ability to affect multiple pathways through the modulation of a single miRNA and that many of the hallmarks of cancer are driven by miRNAs. 

The use of miRNAs as an epigenetic treatment option is another promising avenue, and can be used by itself or in combination with other therapies for breast cancer. However, the main challenges of utilizing miRNAs in the clinical setting include overcoming poor intrinsic in vivo stability as well as efficient and specific delivery of the miRNA to the tumor site. To address these deficiencies more innovative delivery systems are needed. Both viral vectors and nonviral-based delivery systems can be utilized to circumvent these barriers, however the toxicity and immunogenicity limit the use of viral vectors in the clinic. Therefore, nonviral-based delivery systems gained traction as a promising approach for therapeutic miRNA delivery because of the ease of control over composition, manufacturing, modification, as well as a tolerance for cargo size and lower immunogenicity [161,162]. Particularly, cationic materials that condense negatively charged nucleic acids through electrostatic interactions have shown the efficiency and specificity needed to successfully utilize miRNAs in the clinic. However, due to a relatively lower efficiency than viral vectors, recent research has aimed to improve this by modifying particle size and surface composition (for examples see [163,164,165,166]). 

## 5. Conclusions and Future Perspectives

The recent studies demonstrating that miRNAs target the machinery of the breast epigenome have enhanced our understanding of the crucial roles that miRNAs play in inherited gene expression. A comprehensive list of currently validated epi-miRs and epi-miR targets in breast cancer is shown in Table 2. Although direct targeting of some histone acetyltransferases were found to promote tumor progression, the current data generally support the conclusion that epi-miRs act as tumor suppressors in breast cancer.

Future work will need to identify the direct targets and mechanisms of epi-miRs. This will not only expand the potential targets for therapy and increase their appeal as therapeutics and biomarkers themselves, but also stand to determine in which context the miRNAs act as tumor suppressors or oncogenes (for example miR-22 drives breast cancer progression by regulating the TET family of DNA demethylases [70], but inhibits progression by targeting histone demethylases [115] and deacetylases [116]). In addition to determining miRNA targets, we also need to determine the effects that current epigenetic drugs have on the expression of miRNAs. Some studies suggest that epigenetic drugs can exert antitumor effects by both turning on tumor suppressors as well as miRNAs that target oncogenic mRNAs. Additionally, although significant advances have been made in our understanding of how to deliver miRNA, more work needs to be done on how to efficiently and safely deliver miRNAs to the target site. Thus, by completing this work we will have a better understanding of the role that miRNAs play in regulating epigenetics, and will help to drive miRNAs into the clinic as the next generation of cancer therapy. Overall, the wide range of interactions between miRNAs and the epigenome in breast cancer provides new challenges and opportunities for the development of new therapeutic strategies for the treatment of cancer.

## Figures and Tables

**Table 1:** Mammalian epigenetic modifiers.

Function	Enzyme (Alias)	
DNA methyltransferase	DNMT1	DNMT3B
	DNMT2	DNMT3L
	DNMT3A	
DNA demethylases	TET1	
	TET2	
	TET3	
Histone methyltransferase	KMT1A (SUV39H1)	KMT3E (SMYD3)
	KMT1B (SUV39H2)	KMT3F (NSD3)
	KMT1C (EHMT2)	KMT3G (NSD2)
	KMT1D (EHMT1)	KMT4 (DOT1L)
	KMT1E (SETDB1)	KMT5A (SETD8)
	KMT1F (SETDB2)	KMT5B (SUV420H1)
	KMT2A (MLL)	KMT5C (SUV420H2)
	KMT2B (MLL2)	KMT6A (EZH2)
	KMT2C (MLL3)	KMT6B (EZH1)
	KMT2D (MLL4)	KMT7 (SETD7)
	KMT2E (MLL5)	KMT8A (PRDM2)
	KMT2F (SETD1A)	KMT8B (PRDM9)
	KMT2G (SETD1B)	KMT8C (PRDM6)
	KMT2H (ASH1L)	KMT8D (PRDM8)
	KMT3A (SETD2)	KMT8E (MECOM)
	KMT3B (NSD1)	KMT8F (PRDM16)
	KMT3C (SMYD2)	SMYD4 (ZMYND21)
	KMT3D (SMYD1)	
Histone demethylase	KDM1A (LSD1)	KDM5B (JARID1B)
	KDM1B (LSD2)	KDM5C (JARID1C)
	KDM2A (FBXL11)	KDM5D (JARID1D)
	KDM2B (FBXL10)	KDM6A (UTX)
	KDM3A (JMJD1A)	KDM6B (JMJD3)
	KDM3B (JMJD1B)	KDM6C (UTY)
	KDM3C (JMJD1C)	KDM7A (JHDM1D)
	KDM4A (JMJD2A)	KDM7B (PHF8)
	KDM4B (JMJD2B)	KDM7C (PHF2)
	KDM4C (JMJD2C)	KDM8 (JMJD5)
	KDM4D (JMJD2D)	JMJD6
	KDM4E (JMJD2E)	PADI4
	KDM4F (JMJD2F)	NO66
	KDM5A (JARID1A)	
Histone acetyltransferase	KAT1 (HAT1)	KAT7 (HBO1, MYST2)
	KAT2A (GCN5)	KAT8 (hMOF, MYST1)
	KAT2B (PCAF)	KAT9 (ELP3)
	KAT3A (CREBBP)	KAT12 (GTF3C4)
	KAT3B (EP300)	KAT13A (NCOA1)
	HAT4 (NAA60)	KAT13B (NCOA3)
	KAT4 (TAF1)	KAT13C (NCOA2)
	KAT5 (TIP60)	KAT13D (CLOCK)
	KAT6A (hMOZ, MYST3)	KAT14 (CRP2BP)
	KAT6B (hMORF, MYST4)	MCM3AP (GANP)
Histone deacetylase	HDAC1	HDAC10
	HDAC2	HDAC11
	HDAC3	SIRT1
	HDAC4	SIRT2
	HDAC5	SIRT3
	HDAC6	SIRT4
	HDAC7	SIRT5
	HDAC8	SIRT6
	HDAC9	SIRT7

**Table 2:** Epigenome regulators targeted by microRNAs in breast cancer.

microRNA	miRNA acts as a Tumor Suppressor or Oncogenic miRNA (OncomiR)	Target (alias)	Function of Target in Citation	Reference
miR-7	Tumor suppressor miRNA	KMT1E (SETDB1)	Promotes STAT3 expression which drives c-MYC, TWIST, and miR-9 expression in BCSCs	[107]
miR-7	Tumor suppressor miRNA	KMT5A (SETD8)	Drives DNA double stranded breaks and promotes DNA repair	[108]
miR-10b	OncomiR	HDAC4	Promotes tamoxifen sensitivity; enhances tamoxifen-induced apoptosis	[109]
miR-17	Tumor suppressor miRNA	KAT13B (NCOA3)	Promotes taxol resistance by increasing Bcl-2 expression	[110]
miR-17-5p	Tumor suppressor miRNA	KAT13B (NCOA3)	Accelerates proliferation; enhances E2F1- and estrogen receptor-mediated gene expression	[111]
miR-17-5p	Tumor suppressor miRNA	KAT13B (NCOA3)	Accelerates proliferation; enhances estrogen receptor-mediated gene expression	[112]
miR-20b	Tumor suppressor miRNA	KAT13B (NCOA3)	Promotes taxol resistance by increasing Bcl-2 expression	[110]
miR-22	OncomiR	HDAC4	Transcriptional repressor of p21 and p27; Regulates sensitivity to fulvestrant	[113]
miR-22	OncomiR	KAT5 (TIP60)	Involved in the inhibition of EMT; Decreases cell migration and invasion	[114]
miR-22	Tumor suppressor miRNA	KDM7B (PHF8)	Promotes EMT; Binds to transcriptional start site of, and activates, *SNAI1*; Drives cell proliferation, migration, and tumor growth	[115]
miR-22	Tumor suppressor miRNA	SIRT1	Promotes proliferation, migration, and invasion; Inhibits senescence	[116,117]
miR-22	OncomiR	TET1	Promotes global 5hmC levels	[70]
miR-22	OncomiR	TET2	Promotes global 5hmC levels; Inhibits cell migration, EMT, and mammosphere formation; Promotes miR-200 family expression	[70]
miR-22	OncomiR	TET3	Promotes global 5hmC levels; Inhibits cell migration, EMT, and mammosphere formation; Promotes miR-200 family expression	[70]
miR-23a	Tumor suppressor miRNA	KDM4A (JMJD2A)	Promotes transient site-specific copy gains; Drives drug resistance through expression of *CKS1B*	[92]
miR-23b	Tumor suppressor miRNA	KDM4A (JMJD2A)	Promotes transient site-specific copy gains	[92]
miR-25	OncomiR	KAT3B (EP300)	Inhibits EMT through increased E-cadherin expression; Blocks drug- and irradiation-induced senescence	[118]
miR-26a	Tumor suppressor miRNA	KMT6A (EZH2)	Inhibits apoptosis; Promotes anchorage-independent growth and tumorigenesis	[87]
miR-26b	Tumor suppressor miRNA	KMT6A (EZH2)	Promotes tumor aggressiveness and the inflammatory phenotype	[88]
miR-29a	OncomiR	TET1	Inhibits cell proliferation, migration, and EMT	[71]
miR-29b	OncomiR	TET1	Inhibits cell proliferation, anchorage-independent growth, migration, and EMT; Binds to the promoter region of the ZEB2	[72]
miR-29b-5p	Tumor suppressor miRNA	KAT13B (NCOA3)	Accelerates proliferation; enhances estrogen receptor-mediated gene expression	[112]
miR-34a	Tumor suppressor miRNA	HDAC1	Deacetylates HSP70 K246; Promotes cancer cell survival and drug resistance by inhibiting autophagic cell death	[119]
miR-34a	Tumor suppressor miRNA	HDAC7	Deacetylates HSP70 K246; Promotes cancer cell survival and drug resistance by inhibiting autophagic cell death	[119]
miR-34a	Tumor suppressor miRNA	SIRT1	Promotes expansion of BCSCs; Drives tumor growth	[120]
miR-92b	Tumor suppressor miRNA	KMT6A (EZH2)	Blocks rapamycin-induced autophagy; Enhances cell viability and invasion	[89]
miR-93	OncomiR	KAT3B (EP300)	Inhibits EMT through increased E-cadherin expression; Blocks drug- and irradiation-induced senescence	[118]
miR-101	Tumor suppressor miRNA	KMT6A (EZH2)	Drives a metastatic tumor phenotype, oncogenic gene expression, and cell invasion	[121]
miR-106a	Tumor suppressor miRNA	KAT13B (NCOA3)	Accelerates proliferation; enhances estrogen receptor-mediated gene expression	[112]
miR-106b	Tumor suppressor miRNA	KAT13B (NCOA3)	Accelerates proliferation; enhances estrogen receptor-mediated gene expression	[112]
miR-106b	OncomiR	KAT3B (EP300)	Inhibits EMT through increased E-cadherin expression; Blocks drug- and irradiation-induced senescence	[118]
miR-125a-5p	Tumor suppressor miRNA	HDAC4	Promotes cell proliferation, migration, and invasion; Decreases expression of HDAC5 and HDAC7	[122]
miR-125a-5p	Tumor suppressor miRNA	HDAC5	Drives cell proliferation, migration, and invasion; Decreases apoptosis by deacetylating RUNX3 and reduces RUNX3-p300 complex binding to target promoters	[123]
miR-128	Tumor suppressor miRNA	SIRT1	Deacetylates p53 and suppresses its transcriptional targets; Increases Akt signaling; Protects against apoptosis by decreasing PUMA expression	[124]
miR-137	Tumor suppressor miRNA	KAT13A (NCOA1)	Accelerates proliferation; Decreases cell viability	[112]
miR-137	Tumor suppressor miRNA	KAT13B (NCOA3)	Accelerates proliferation; enhances estrogen receptor-mediated gene expression	[112]
miR-137	Tumor suppressor miRNA	KAT13C (NCOA2)	Accelerates proliferation; Decreases cell viability	[112]
miR-137	Tumor suppressor miRNA	KDM4A (JMJD2A)	Promotes transient site-specific copy gains	[92]
miR-137	Tumor suppressor miRNA	KDM5B (JARID1B)	Drives cell proliferation and migration	[94]
miR-138	Tumor suppressor miRNA	KDM5C (JARID1C)	Drives cell proliferation	[94]
miR-138	Tumor suppressor miRNA	KDM6B (JMJD3)	Promotes cell proliferation, invasion, and EMT through H3K27me3 demethylation	[125]
miR-138	Tumor suppressor miRNA	KMT6A (EZH2)	Drives cell invasion EMT, and primary tumor growth	[126]
miR-143	Tumor suppressor miRNA	DNMT3A	Accelerates cell proliferation and anchorage-independent growth; Hypermethylates *PTEN* and *TNFRSF10C*	[52]
miR-148	n/a	DNMT3B	n/a; Only studied binding of miR-148 of different DNMT3B splice variants in normal and cancer cells	[54]
miR-148a	Tumor suppressor miRNA	DNMT1	Promotes cell proliferation, colony formation, and tumor angiogenesis; Drives IGF-IR and IRS1 expression, and promotes Akt and ERK signaling by hypermethylating *miR-148a*; Suppresses *ER-α* expression	[127,128]
miR-152	Tumor suppressor miRNA	DNMT1	Promotes cell proliferation, colony formation, and tumor angiogenesis; Drives IGF-IR and IRS1 expression, and promotes Akt and ERK signaling by hypermethylating *miR-152*	[127]
miR-185	Tumor suppressor miRNA	DNMT1	Drives cell proliferation; Silences BRCA1 expression by hypermethylation	[129]
miR-199	Tumor suppressor miRNA	KMT6A (EZH2)	Accelerates cell proliferation and promotes invasion	[86]
miR-200a	Tumor suppressor miRNA	SIRT1	Promotes cellular transformation, anchorage-independent growth and migration; Binds to miR-200a promoter and recruits DNMT1, 3A, and 3B to suppress expression	[130]
miR-200b	Tumor suppressor miRNA	DNMT3A	Binds, along with c-MYC, to the miR-200b promoter to silence expression and drive EMT	[53]
miR-200b	Tumor suppressor miRNA	KAT13B (NCOA3)	Accelerates proliferation; enhances estrogen receptor-mediated gene expression	[112]
miR-200b	Tumor suppressor miRNA	SUZ12	Promotes formation and maintenance of mammospheres, as well as tumor growth and the maintenance of CSCs through H3K27me3 and recruitment of PcG suppression of *E-cadherin*	[131]
miR-200c	Tumor suppressor miRNA	KAT13B (NCOA3)	Accelerates proliferation; enhances estrogen receptor-mediated gene expression	[112]
miR-211-5p	Tumor suppressor miRNA	SETBP1	Promotes cell proliferation, invasion, and migration, as well as metastasis	[132]
miR-214	Tumor suppressor miRNA	KMT6A (EZH2)	Accelerates cell proliferation and promotes invasion	[86]
miR-221	OncomiR	DNMT3B	Inhibits stemness by repressing Nanog and Oct3/4 expression	[51]
miR-222	OncomiR	DNMT3B	Inhibits stemness by repressing Nanog and Oct3/4 expression	[51]
miR-340	Tumor suppressor miRNA	KMT6 (EZH2)	Drives cell proliferation, invasion, migration, and induces apoptosis via DNMT1 expression and decreased miR-200a/b expression	[133]
miR-342	OncomiR	DNMT1	Binds to *ID4* promoter for normal breast function and development	[134]
miR-381-3p	Tumor suppressor miRNA	KMT1E (SETDB1)	Drives cell proliferation, cell cycle progression and migration; Accelerates primary tumor growth	[135]
miR-448	Tumor suppressor miRNA	KDM5B (JARID1B)	Enhances proliferation, anchorage-independent growth, migration, invasion, and promotes a stem-like phenotype via induction of lncRNA MALAT1	[93]
miR-491-5p	Tumor suppressor miRNA	KDM4B (JMJD2B)	Suppresses estrogen receptor-mediated signaling and cell proliferation	[136]
miR-502	Tumor suppressor miRNA	KMT5A (SETD8)	Promotes cell cycle progression, migration, invasion, and EMT	[137]
miR-519d	Tumor suppressor miRNA	KAT13B (NCOA3)	Accelerates proliferation; enhances estrogen receptor-mediated gene expression	[112]
miR-590-3p	Tumor suppressor miRNA	SIRT1	Drives cell survival; Inhibits apoptosis; Prevents acetylation and activation of p53	[138]
miR-708	Tumor suppressor miRNA	KDM1A (LSD1)	Promotes cell proliferation and invasion	[91]
miR-1307-3p	OncomiR	SMYD4	Suppresses cell proliferation and anchorage-independent growth; Inhibits tumor formation	[139]
miR-1915-3p	Tumor suppressor miRNA	KMT2F (SETD1A)	Drives estrogen receptor-mediated signaling through H3K4 methylation; Enhances cell proliferation and migration; Suppresses apoptosis	[140]
miR-3666	Tumor suppressor miRNA	SIRT7	Promotes cell proliferation; Inhibits apoptosis	[141]
